# Effects of positioning on radiographic measurements of ankle morphology: a computerized tomography-based simulation study

**DOI:** 10.1186/1475-925X-12-131

**Published:** 2013-12-20

**Authors:** Chien-Chung Kuo, Hsuan-Lun Lu, Tung-Wu Lu, Cheng-Chung Lin, Alberto Leardini, Mei-Ying Kuo, Horng-Chaung Hsu

**Affiliations:** 1 Institute of Biomedical Engineering, National Taiwan University, No. 1, Sec. 1, Jen-Ai Road, Taipei 100, Taiwan, ROC; 2 Department of Orthopedics, China Medical University Hospital, Taichung, Taiwan, ROC; 3 Department of Orthopedic Surgery, School of Medicine, National Taiwan University, Taipei, Taiwan, ROC; 4 Movement Analysis Laboratory, Istituto Ortopedico Rizzoli, Bologna, Italy; 5 Department of Physical Therapy, China Medical University, Taichung, Taiwan, ROC

**Keywords:** Simulated radiograph, Talus, Tibia, Reliability, Sensitivity analysis

## Abstract

**Background:**

Measurements of the morphology of the ankle joint, performed mostly for surgical planning of total ankle arthroplasty and for collecting data for total ankle prosthesis design, are often made on planar radiographs, and therefore can be very sensitive to the positioning of the joint during imaging. The current study aimed to compare ankle morphological measurements using CT-generated 2D images with gold standard values obtained from 3D CT data; to determine the sensitivity of the 2D measurements to mal-positioning of the ankle during imaging; and to quantify the repeatability of the 2D measurements under simulated positioning conditions involving random errors.

**Method:**

Fifty-eight cadaveric ankles fixed in the neutral joint position (standard pose) were CT scanned, and the data were used to simulate lateral and frontal radiographs under various positioning conditions using digitally reconstructed radiographs (DRR).

**Results and discussion:**

In the standard pose for imaging, most ankle morphometric parameters measured using 2D images were highly correlated (R > 0.8) to the gold standard values defined by the 3D CT data. For measurements made on the lateral views, the only parameters sensitive to rotational pose errors were longitudinal distances between the most anterior and the most posterior points of the tibial mortise and the tibial profile, which have important implications for determining the optimal cutting level of the bone during arthroplasty. Measurements of the trochlea tali width on the frontal views underestimated the standard values by up to 31.2%, with only a moderate reliability, suggesting that pre-surgical evaluations based on the trochlea tali width should be made with caution in order to avoid inappropriate selection of prosthesis sizes.

**Conclusions:**

While highly correlated with 3D morphological measurements, some 2D measurements were affected by the bone poses in space during imaging, which may affect surgical decision-making in total ankle arthroplasty, including the amount of bone resection and the selection of the implant sizes. The linear regression equations for the relationship between 2D and 3D measurements will be helpful for correcting the errors in 2D morphometric measurements for clinical applications.

## Background

Total ankle arthroplasty (TAA) is an important treatment option for the management of advanced ankle osteoarthritis, a disease often leading to impairment of locomotion, physical disability, and reduced quality of life [1,2]. Clinical success of TAA depends heavily on the available information on the morphology of the relevant bones, which is critical for the design of ankle prostheses and for the procedures of their surgical implantation [3,4]. Restoration of the ankle joint using TAA based on anatomical dimensions has been suggested to lead to the best clinical results [5-7].

It is well recognized that conformity of a TAA design to the bone morphology, including the proper sizing of the components, is an important factor for the success of the prosthesis in replicating the function of the joint [8]. For example, the radius of curvature of the talar component is critical for the compatibility of the total ankle prosthesis with the dimensions of the ligaments, affecting the mobility and the stability of the replaced ankle [3,9,10]. A radius smaller than normal may lead to slackening of ligaments and thus joint instability, while a radius greater than normal could result in joint motion limitation. Using implants of precisely matched sizes can thus substantially reduce complications and increase survival rates [11-14]. A good shape match between the prosthesis and the resected surfaces of the bones is also an important factor for long-term fixation in TAA [12,15,16]. Therefore, errors in the estimation of the patient-specific morphological parameters may have critical effects on the pre-surgical decision-making in TAA, including the selection of the size of the implants.

Among the clinically available medical imaging modalities, bi-planar radiographs are commonly used for this purpose owing to their convenience, low cost, and low radiation dose compared with other modalities such as MRI or CT [17]. Since bi-planar radiographs are based on two-dimensional (2D) projective images, bones at different distances from the projection plane will produce bone images of different size, position and intensity. In addition, the intrinsic articular surfaces of the ankle joint are not symmetrical, and are often oblique with respect to the anatomical planes of the tibia, fibula and talus. It is difficult to describe all the anatomical dimensions in detail with only one radiograph, either in the anteroposterior (A/P), mediolateral (M/L) or mortise (M) view, leading to errors in measurements and interpretations.

For a morphological measurement of the ankle on a radiograph to be accurate, the true positions of anatomical bony landmarks should be identifiable on the radiograph, and the bones have to be positioned correctly with respect to the X-ray source to avoid projecting oblique shapes and overlapping surfaces onto the radiograph, i.e., radiographs of the ankle must be A/P, M/L or M views. However, this ideal positioning is difficult to achieve in clinical practice. The positioning error of the ankle as a result of contact of the exterior shape of the lower limb with the image plane, or improper position of the beam focus by an operator during the radiography procedure might cause artificial projection errors on the planar radiograph [18,19].

Computerized Tomography (CT) scans can be used to obtain more accurate morphological data of the ankle bones [20] than those obtained from 2D images [4,21]. However, since CT scans are expensive and involve relatively high radiation dosage, 2D imaging is still in use clinically for evaluating the ankle and selecting the size of the prosthesis. To the best knowledge of the authors, no study has evaluated quantitatively the errors involved in the morphological measurements using 2D imaging methods, which may have an impact on the diagnosis, treatment planning, and the outcomes. By taking advantage of computer simulation, measurement errors in 2D imaging may be obtained by comparing 2D and 3D measurements. This will help clarify whether 2D image-based measurement errors are affected by bone poses in space during imaging, and whether they can be used to find the calibration relationship between measurements based on 2D images and 3D reconstruction for clinical applications.

The purposes of this study were (a) to compare the measurements of ankle morphology in the standard pose using 3D CT images with those using 2D images, and to determine the relationships between 2D and 3D measurements using linear regression analysis; (b) to determine the sensitivity of the 2D measurements to the errors associated with the mal-positioning of the ankle during imaging; (c) to quantify the repeatability of the 2D measurements under simulated positioning conditions involving random errors; and (d) to evaluate the performance of the linear regression equations obtained in (a) in predicting 3D measurements from 2D measurements.

## Methods

### Overview

**Figure 1:**
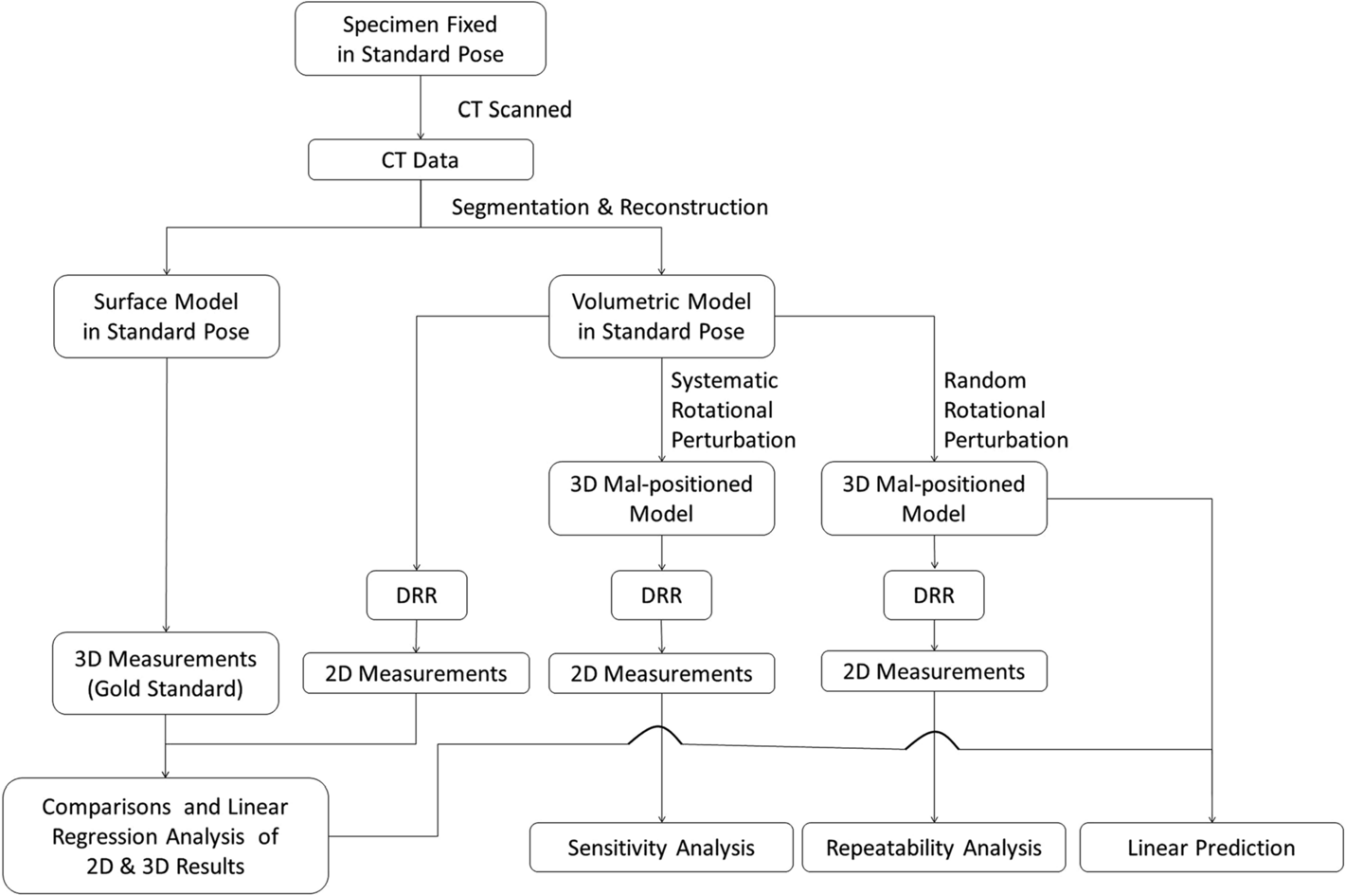
**Procedural framework of the study.** The general procedural framework for the current study, indicating the steps of the experiments and computer simulations for the three objectives of the study, namely comparisons of 2D and 3D measurements, sensitivity analysis and repeatability analysis of 2D measurements.

The general procedural framework for the current study is shown in Figure 1, indicating the steps of the experiments and computer simulations for the three objectives of the study, namely comparisons and linear regression analysis of 2D and 3D measurements, sensitivity analysis and repeatability analysis of 2D measurements. Cadaveric ankles were CT scanned and the data were used to reconstruct surface and volumetric models of the bones, which were then used to simulate sagittal and frontal radiographs using digitally reconstructed radiographs (DRR). Three-dimensional morphological measurements were performed on the 3D CT-derived bone models and taken as the gold standard. Two-dimensional measurements were performed on the CT-generated planar DRR images. Comparisons between 3D and 2D measurements enabled the assessment of errors in 2D measurements and the determination of their correlations, while comparisons of 2D measurements between standard pose and perturbed poses were used for sensitivity and repeatability analysis of measurements based on 2D images (Figure 1).

### Specimen preparation and CT scan

**Figure 2:**
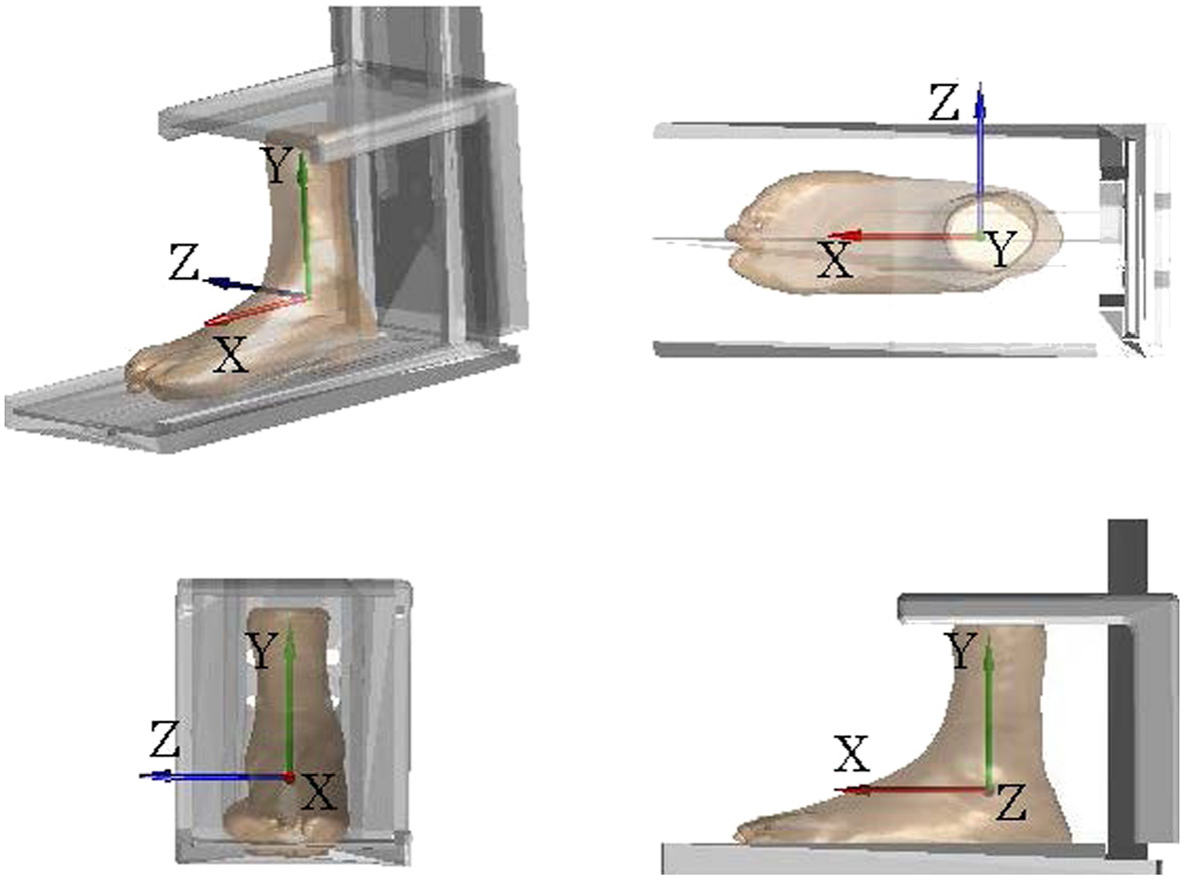
**The global coordinate system of the ankle specimen.** The fixation of the ankle specimen in the standard pose for imaging. The global coordinate system of the ankle specimen was defined with the origin at the geometric center of the talus. The anteroposterior (X) axis was defined as the line joining the calcaneal insertion of the Achilles tendon and the head of the second metatarsal. The superoinferior (Y) axis was defined as the tibial longitudinal axis which was perpendicular to the base-plate. The mediolateral (Z) axis was then defined as the line perpendicular to both the X- and Y-axes.

Fifty-eight fresh frozen ankle specimens, 22 females and 36 males, were used in the current study (Table 1). These specimens were obtained from donors who had undergone below-knee amputation procedures for reasons other than trauma or disease of the ankle joint. The specimens were stored at -70°C immediately after harvest and thawed at room temperature 24 hours prior to experiment. Each ankle specimen was positioned in the neutral joint position, i.e., standard pose*,* in a plastic frame (Figure 2), according to previously determined procedures [20]. The specimen was fixed to a base-plate using bone cement, with the long axis of the base-plate of the frame aligned with the line joining the calcaneal insertion of the Achilles tendon and the second metatarsal head. The standard pose of the ankle was then defined when the longitudinal axis of the shank was perpendicular to the base-plate as indicated by a goniometer. The spine of the plastic frame was adjusted to accommodate specimens with different lengths of the remaining shank. The proximal ends of the shank bones were then fixed to the upper plate using bone cement. This procedure enabled a reliable definition of the anatomical frame for the specimen as a whole without the potential errors associated with the difficultly of identifying bony landmarks [20]. After fixation, the specimen-fixation construct was scanned with a 16-slice spiral CT scanner (GE BrightSpeed16, C&G Technologies, USA) with a slice thickness of 0.625 mm. The resolution of the obtained CT images was 512 × 512 (pixels) and the voxel size was 0.630 × 0.630 × 0.625 (mm^3^).

**Table 1:** Demographic data of the donors of the ankle specimens

	**Mean (SD)**	**Min**	**Max**
Foot length (cm)	22.0 (1.7)	19.0	25.5
Age at donation (years old)	65.3 (11.7)	40	87
Height (cm)	163.3 (8.8)	142	180
Body mass (kg)	65.4 (12.4)	44	95

Demographic data of the 58 donors of the ankle specimens, 36 males and 26 females. These specimens were obtained from below-knee amputation procedures undergone for reasons other than trauma or disease of the ankle joint.

### Bone model reconstruction and morphological parameters

**Figure 3:**
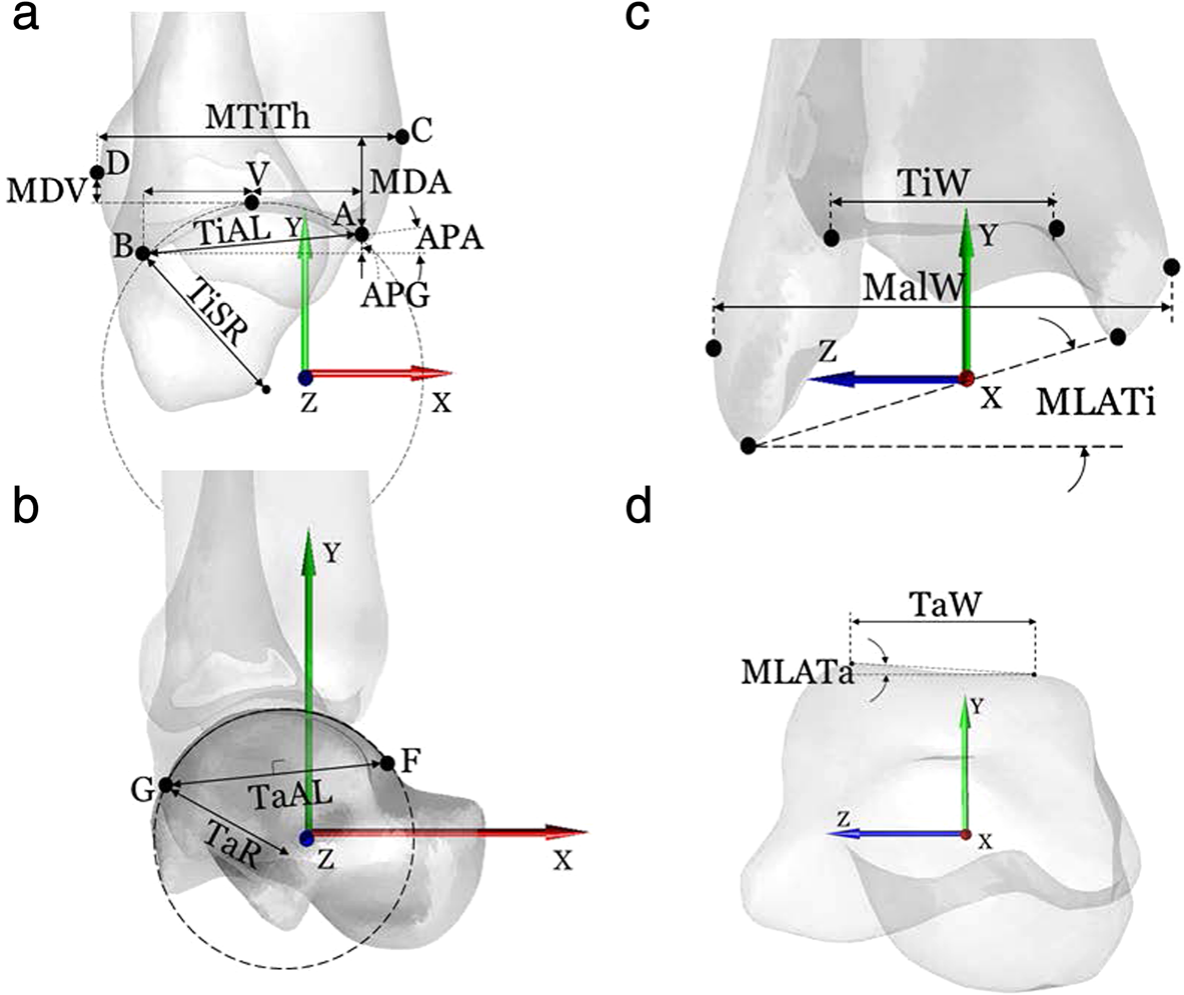
**Definitions of ankle morphological parameters.** Graphical depiction of the parameters defined on the 3D bone models as seen in the sagittal plane **(a-b)** and frontal plane **(c-d)**. Definitions of the parameters are also provided in Table 2.

Two types of 3D models of the bones, i.e., volumetric and surface models, together with the plastic frame were reconstructed from the CT images following Lin *et al*. [22]. The volumetric model of the ankle specimen as a whole (namely the tibia, fibula and talus) were segmented from the CT data set. In addition to the volumetric model, a triangle-meshed surface model of each bone was also reconstructed from the CT slices. All the required image processing for model construction was performed using a commercial software package (AMIRA, Visage Imaging Inc., Germany). A coordinate system was embedded in both models of the ankle specimen with the origin at the geometric center of the talus; the anteroposterior (X) axis parallel to the base-plate, the superoinferior (Y) axis perpendicular to the base-plate, and the mediolateral (Z) axis as the line perpendicular to both the X- and Y-axes. A total of 14 morphological parameters, ten for the tibia-fibula segment and four for the talus, were determined automatically [4,20] for the bone models in the standard pose based on the 3D geometrical definitions given in Figure 3 and Table 2, using an in house-developed program in MATLAB (R2010a, The MathWorks, Inc., USA). The data were then taken as the gold standard values. All the landmarks associated with the morphological parameters were also determined automatically by the software and were verified by an experienced orthopaedic surgeon (CCK) before subsequent use for automatic labeling of the landmarks identified on 2D images.

**Table 2:** Definitions of the ankle morphological parameters

**Distal Tibia**	
TiAL (mm)	*Tibial arc length*: distance between the most anterior (A) and posterior (B) points of the maximal arc of the tibial mortise in the sagittal plane
TiSR (mm)	*Tibial sagittal radius*: radius of the AB arc
APG (mm)	*Anterio-posterior gap*: longitudinal (Y-axis) component of the distance between A and B
APA (deg)	*Anterio-posterior inclination angle*: inclination angle between the X-axis and the AB segment
MTiTh (mm)	*Maximal tibial thickness*: The A/P distance from the most anterior (C) to the most posterior (D) point on the tibial profile in the sagittal plane
MDA (mm)	Longitudinal (S/I) distance between A and C
MDV (mm)	Longitudinal (S/I) distance between the most proximal vertex of the tibial mortise (V) and the point D
TiW (mm)	*Tibial width*: M/L distance of the tibial mortise calculated using the two end-points of the anterior (TiW*a*) and posterior (TiW*p*) edges
MalW (mm)	*Malleolar width*: M/L distance between the most lateral point of the fibula and the most medial point of the tibia
MLATi (deg)	Angle in the frontal plane between the M/L axis and the line joining the most distal points of the fibula and tibia
**Talus**	
TaAL (mm)	*Trochlea tali arc length*: distance between the most anterior (F) and posterior (G) and proximal (H) points of the trochlea tali, as seen in the sagittal projection of the talus. The suffixes *m*, *l* and c indicate the corresponding medial, lateral and central arcs, respectively
TaW (mm)	*Trochlea tali width*: width between medial and lateral crests of the talar dome. The suffix *a, p* and *c* indicates this width, respectively, in the most anterior, most posterior and central location along M/L axis, i.e., of F*m*F_*l*_, G_*m*_G_*l*_ and H_*m*_H_*l*_ segments
TaR (mm)	*Trochlea tali radius*: radius of the talar dome in the sagittal plane, as identified by the arc GF. The suffixes *m*, *l* and *c* indicate the corresponding medial, lateral and central arcs
MLATa (deg)	Angle in the frontal plane between the M/L axis and the line joining the two most proximal vertices of the trochlea tali

Definitions of the parameters used to describe the morphology of the ankle joint. See also Figure 3 for relevant graphical descriptions.

### Generation of digitally reconstructed radiograph (DRR)

**Figure 4:**
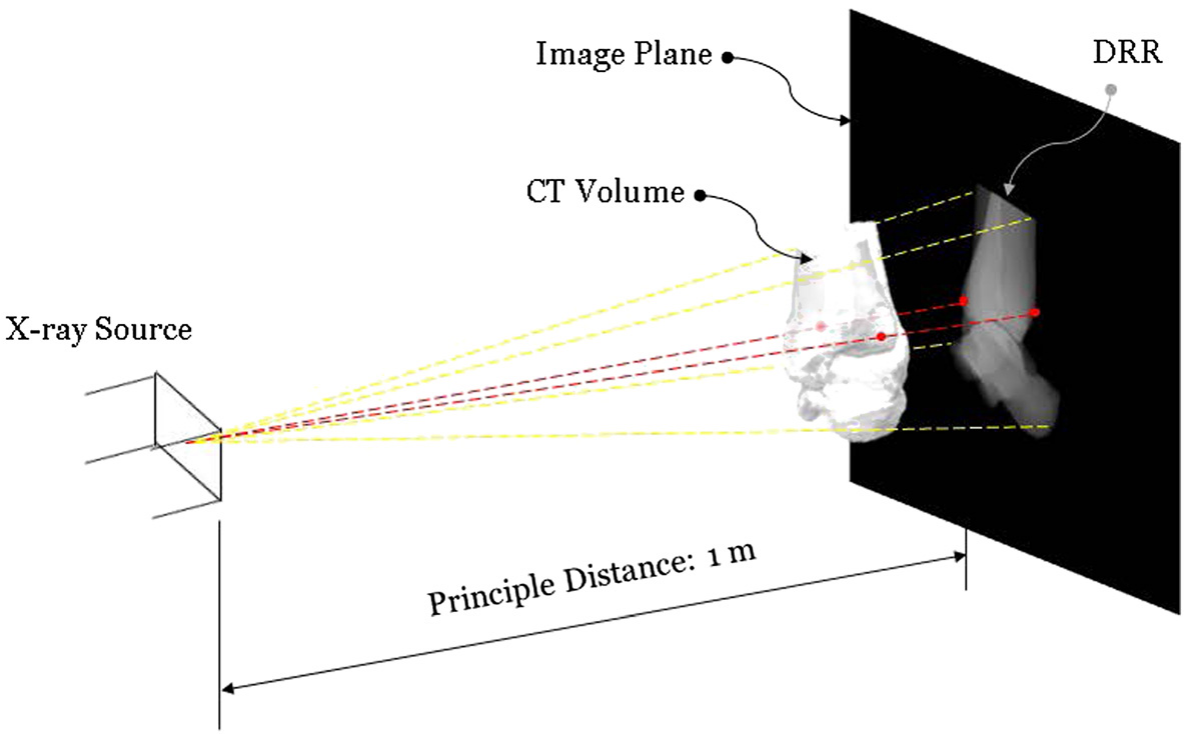
**Projective projection of CT bone data to obtain a Digitally Reconstructed Radiograph (DRR).** The projective projection of the CT bone data was performed simulating the standard X-ray imaging of the ankle on a digital radiography system (CXDI-40EG, CANON, USA) with the X-ray focus 1 meter away from the image plane. The ankle model was positioned in the standard pose with the anatomical axes parallel to those of the X-ray system depending on the direction of the imaging. The DRR of the bone was generated by casting rays from the X-ray source through the volume of the bone model. Each of these rays went through a number of voxels of the volume, the attenuation coefficients of which were then integrated along the ray and projected onto the imaging plane to obtain a DRR image resembling a radiograph. During the generation of the DRR for 2D measurements, the landmarks (red) associated with the morphological parameters determined were also generated automatically using the geometrical features of the 3D volumetric bone models (Table 2), and projected onto the 2D image plane. The projected 3D landmarks were used to label the landmarks automatically using a 2D-to-3D registration procedure [24].

Given the positions of the X-ray source and a CT-derived volumetric bone model in space with respective to the image plane, the DRR of the bone was generated by casting rays through the volume of the bone model [22]. Each of these rays went through a number of voxels of the volume, the attenuation coefficients of which were then integrated along the ray and projected onto the imaging plane to obtain a DRR image resembling a radiograph (Figure 4). In order to reduce the time required for DRR generation, the ray-tracing was implemented with trilinear interpolation in MATLAB (R2010a, The Mathworks, Inc., USA) [23]. In the current study, the DRRs were generated simulating the standard X-ray imaging of the ankle on a digital radiography system (CXDI-40EG, CANON, USA) in which the X-ray focus was 1 meter away from the image plane.

### 2D Measurements in standard pose

Measurements of the morphological parameters on planar radiographs were first performed with the ankle model in the standard pose for imaging, with the anatomical axes parallel with those of the X-ray system depending on the direction of the imaging (Figure 4). For the DRR simulating an M/L radiograph, the ankle model was positioned such that the lateral malleolus was in contact with the image plane and the principal axis passed through the medial malleolus. For the DRR simulating an A/P radiograph, the ankle model was positioned such that the most posterior aspect of the calcaneus was in contact with the image plane and the principal axis passed through the mid-point of the inter-malleolar axis. During the generation of the DRR for 2D measurements, the landmarks associated with the morphological parameters were also generated automatically using the geometrical features of the 3D volumetric bone models (Table 2), and projected onto the 2D image (Figure 4). The values of the morphological parameters on the DRR were calculated following the same definition as in 3D measurements without using the projected 3D landmarks. Some parameters, such as TaR and SRTi, were calculated with edge detection on the DRR image and minimum human involvement. For parameters that are defined by bony landmarks, an experienced orthopaedic surgeon (CCK) identified the landmarks directly on the DRR. The reliability of this procedure was determined by repeated identification of the landmarks by the same surgeon, giving an Intra-Class Correlation coefficient (ICC) of 0.9, which was considered strong for the current purpose. After the manual identification, the projected 3D landmarks were then used to label the landmarks automatically using a 2D-to-3D registration procedure [24] for subsequent analysis. This automatic procedure removed human errors in naming the landmarks. The normality of the parameters was tested using the Shapiro-Wilk test. Statistical comparisons between 2D and 3D measurements for each parameter were performed using a paired t-test, and their association was determined using Pearson’s correlation analysis. Linear regression was also implemented to provide the relation between 2D and 3D measurements.

### Sensitivity analysis of 2D measurements to Mal-positioning

**Figure 5:**
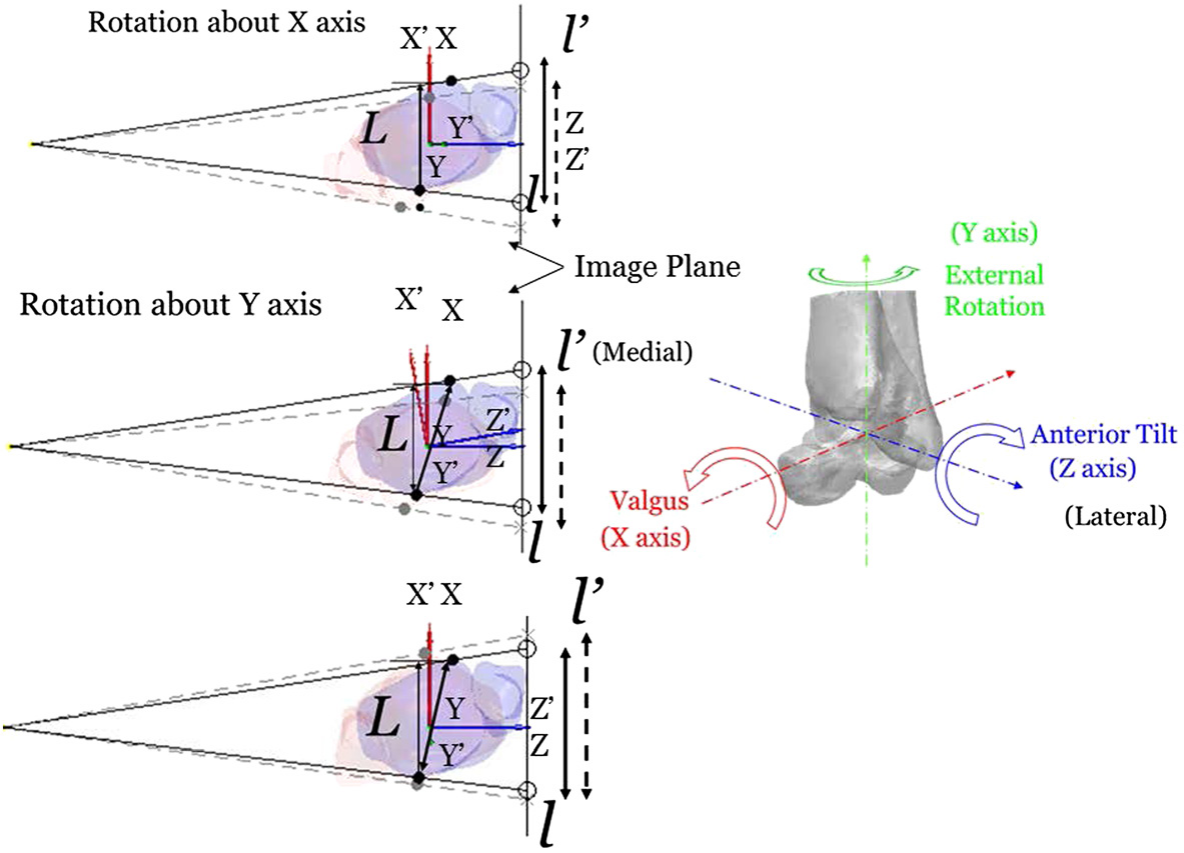
**Sensitivity analysis of the ankle morphological parameters.** Sensitivity of the ankle morphological parameters in response to deviations in the three rotational components away from the standard pose on the M/L radiograph. The ankle model was positioned such that the lateral malleolus was in contact with the image plane with the principal axis passing through the medial malleolus. From the standard pose defined by the coordinate system (X, Y, Z), the ankle model was rotated by 6 degrees at 1-degree intervals in both directions about each of the three anatomical axes to give a perturbed pose as described by the coordinate system (X’, Y’, Z’). As a schematic representation, the landmarks associated with 3D morphological parameters (black: original position of the landmarks; grey: landmark positions after perturbation) are also shown on the 2D image via projection. The distances of the landmarks on 2D image (L: 3D value; *l*: unperturbed landmark distances; *l’*: perturbed landmark distances) varied with different rotational perturbations. A similar perturbation approach was also used for analysis of parameters defined on the A/P radiograph.

A systematic sensitivity analysis was performed to determine the changes in the 2D measurements as a result of small deviations about each of the three anatomical axes of the ankle specimen from the standard pose. From the standard pose, the model of the ankle specimen was then rotated by 6 degrees at 1-degree intervals in both directions about each of the three axes of the coordinate system, respectively (Figure 5). The maximum ranges of the rotations were selected reflecting the possible errors in aligning the ankle joint for X-ray imaging in the clinical protocol currently in use in our hospital, such as those associated with the effects of the profile of the shank during M/L imaging and the size of the calcaneus and the profile of the thigh during A/P imaging. Therefore, for each specimen 2D morphological parameters were measured from 37 DRRs of different specimen model poses (including the standard pose) for each of the M/L and A/P views. The trend of each of the morphometric measurements in relation to the rotational perturbations about each axis of the coordinate system was also determined using a polynomial test.

### Repeatability analysis of 2D measurements

For the repeatability of the 2D measurements, each specimen was perturbed from its standard pose with random errors in the three individual rotational components to obtain ten new poses. The random errors were generated within the above-mentioned maximum ranges. For each specimen 2D morphological parameters were measured from the DRRs of the ten different specimen model poses for each of the M/L and A/P views. Repeatability of the measurements of each parameter was then assessed in terms of the Intra-Class Correlation coefficients using a 2-way mixed-effects average model (ICC3, k) for intra-examiner assessment [25]. The values of the ICC ranging from 0.81 to 1.0 indicated very good reliability; 0.61–0.80 good; 0.41–0.60 moderate; 0.21–0.40 fair; and below 0.2 poor reliability [26].

### Prediction of 3D measurements using 2D measurements via linear regression equations

For evaluating the performance of the linear regression equations derived from data in the standard pose for predicting the 3D measurements from 2D measurements, each specimen was perturbed from its standard pose with random errors in the three individual rotational components to obtain two sets of ten new poses, one set with random errors within a maximum of 3 degrees and another set within 6 degrees. The DRRs of these perturbed poses in the M/L and A/P views were used to obtain 2D measurements of the morphological parameters. These measurements were then corrected using the corresponding linear regression equations to give corrected 2D measurements. The residual errors of the corrected 2D measurements were calculated by comparing them to the 3D measurements. All the statistical analysis was performed using SPSS 13.0 (SPSS Inc., Chicago, USA).

## Results

The results of the Shapiro-Wilk test showed that the parameters all had a normal distribution. In the standard pose without any positioning errors, most morphological parameters were significantly different between 2D and 3D measurements, except APA, MTiTh, MDV, TaR, MLATi and TaW (Table 3). However, most parameters showed a high correlation between 2D and 3D measurements with correlation coefficients greater than 0.8, and the regression lines showed a slope close to unity (Table 3). Among the parameters showing no significant 2D-3D differences, MTiTh (0.54), MDV (0.37) and TaRm (0.09) had only a moderate to low correlation (Table 3).

**Table 3:** Comparisons between 2D and 3D measurements

		**3D Mean value (SD)**	**2D Mean value (SD)**	Error (%)^*^	***P*-value**	**R**	a^**^	b^**^
**Sagittal Plane**	*Tibia Part*							
	TiAL (mm)	28.41 (2.62)	29.35 (2.75)	3.28	<0.001	1.00	0.95	0.39
	TiSR (mm)	29.07 (8.30)	30.04 (8.66)	3.32	<0.001	1.00	0.96	0.25
	APG (mm)	3.85 (2.65)	3.98 (2.74)	3.18	<0.001	1.00	0.97	-0.01
	APA (deg)	7.86 (5.40)	7.86 (5.40)	0.00	0.86	1.00	1.00	0.00
	MTiTh (mm)	42.44 (4.83)	44.64 (3.07)	5.17	0.10	0.54	0.72	10.22
	MDA (mm)	10.21 (2.47)	12.68 (2.99)	24.20	<0.001	0.82	0.81	0.16
	MDV (mm)	3.15 (1.64)	3.11 (1.89)	-1.24	0.11	0.37	0.44	2.01
	*Talus Part*							
	TaAL (mm)	33.55 (4.54)	31.80 (5.29)	-5.22	<0.001	0.81	0.58	15.13
	TaR (mm)	20.57 (2.78)	21.20 (3.13)	3.05	0.57	0.09	0.16	17.06
**Frontal Plane**	*Tibia part*							
	TiW (mm)	32.84 (2.72)	33.95 (2.69)	3.38	<0.001	0.86	0.85	3.96
	MalW (mm)	62.60 (3.69)	63.73 (3.83)	1.80	<0.001	1.00	0.97	0.95
	MLATi (deg)	12.59 (3.08)	12.61 (3.08)	0.13	0.63	0.99	1.00	-0.03
	*Talus part*							
	TaW (mm)	19.89 (4.45)	20.02 (3.82)	0.68	0.34	1.00	0.92	1.89
	MLATa (deg)	2.31 (3.67)	2.06 (3.20)	-10.87	0.01	0.99	1.07	0.04

*Error = 100% × (2D - 3D)/3D.

**Linear Regression: (3D) = a × (2D) + b.

Mean values and standard deviations of 2D and 3D measurements, and the mean errors of 2D measurements as percentages of 3D measurements. Negative error values indicate that the parameter measured from 2D images under-estimated those measured from 3D CT data. Pearson’s correlation coefficients and *P*-values of comparisons between 2D and 3D measurements using a paired t-test are also given. The coefficients (a, b) corresponding to the linear regression analysis on the 2D and 3D measurements are listed.

Further systematic sensitivity analysis showed how the 2D measurements were affected by errors in the mal-positioning in each anatomical plane. With a rotational positioning error of 6 degrees in both directions about the X-axis, most parameters, except TaW, MLATi and MLATa, were not greatly affected, with measurement errors smaller than 10% of the standard values (i.e., those obtained in the standard pose) (Table 4). For those with higher measurement errors, the sensitivities to positive and negative pose perturbations were largely different. The MLATa parameter was the most sensitive, overestimating the standard values by 160.1% for positive rotational perturbations about the X-axis and by 35.6% for negative rotational perturbations. The second most sensitive parameter was MLATi, underestimating the standard values by 36.1% for positive rotational perturbations about the X-axis, and overestimating by 35.3% for negative rotational perturbations. Another sensitive parameter TaW underestimated the standard values by 31.2% for positive rotational perturbations, but by only 18.4% for negative rotational perturbations (Table 4).

**Table 4:** Sensitivity of the 2D measurements to the mal-positioning of the ankle

		**X-axis**							**Y-axis**							**Z-axis**					
		***-6 deg***	***-3 deg***	***Trend***		***3 deg***	***6 deg***		***-6 deg***	***-3 deg***	***Trend***		***3 deg***	***6 deg***		***-6 deg***	***-3 deg***	***Trend***		***3 deg***	***6 deg***
**Sagittal Plane**	*Tibia Part*																				
	TiAL (mm)	0.0	0.0	**-**	**-**	0.0	0.1		1.2	0.6			0.3	0.8		0.0	0.0	**-**	**-**	0.0	0.0
	SRTi (mm)	-0.6	-0.2			-0.1	-0.4		1.4	-0.2			0.0	1.9		0.0	0.0	**-**	**-**	0.0	0.0
	APG (mm)	1.4	0.1	**↖**	**↗**	0.3	0.8		0.5	0.5	**↖**	**↘**	-0.2	-1.4		0.0	0.0	**-**	**-**	0.0	0.0
	APA (deg)	1.3	0.1	**↖**	**↗**	0.2	0.8		-0.1	0.7	**-**	**↘**	-0.1	-1.6		0.0	0.0	**-**	**-**	0.0	0.0
	MTiTh (mm)	-0.1	-0.1	**-**	**-**	0.1	0.2		-1.0	-0.5	**↙**	**↗**	0.4	1.1		-0.7	-0.1			-0.1	-0.4
	MDA (mm)	1.1	0.2			-0.5	-1.3		-0.5	0.4	**-**		-0.2	-2.5		-19.3	-2.0			34.9	56.3
	MDV (mm)	-0.8	-0.6		**↘**	0.5	1.2		-0.7	-0.5			-0.1	-1.6		21.3	1.5			-5.9	-17.2
	*Talus Part*																				
	TaAL (mm)	1.8	0.8	**↖**	**↘**	-0.8	-1.3		1.3	0.7	**↖**	**↘**	-1.1	-2.8		0.0	0.0	**-**	**-**	-0.1	-0.1
	TaR (mm)	1.0	0.6		**↘**	-0.5	-0.5		3.5	1.7	**↖**	**↘**	-1.8	-2.7		0.1	0.0	**-**	**-**	-0.1	-0.2
**Frontal Plane**	*Tibia Part*																				
	TiW (mm)	0.0	0.0	**-**	**-**	0.0	0.0		-0.2	-0.3	**-**	**↗**	0.8	1.8		-0.2	-0.1	**-**	**-**	0.1	0.2
	MalW (mm)	1.1	0.3	**↖**	**↗**	0.5	1.0		1.8	1.0	**↖**	**↘**	-1.0	-2.2		0.0	-0.1	**-**		2.5	2.2
	MLATi (deg)	35.3	18.7	**↖**	**↘**	-17.9	-36.1		-2.4	-1.3	**↙**	**↗**	1.6	3.6		-0.1	0.0	**-**	**-**	-0.3	-0.2
	*Talus Part*																				
	TaW (mm)	-18.4	1.1			2.5	-31.2		1.1	0.7	**↖**	**↘**	-1.0	-2.2		1.9	1.2	**↖**	**↘**	-0.6	-3.0
	MLATa (deg)	35.6	2.1			-11.6	160.1		0.1	-0.7			1.2	2.3		87.0	15.8			31.3	145.3

Sensitivity of the parameters measured from 2D images subject to deviations in rotational components from the standard pose about each of the anatomical axes, as percentages of the values of the parameters measured in the standard pose. Positive and negative values indicated that the deviation caused an over-estimation and an under-estimation of the percentage values of the standard pose, respectively (X-axis: A/P; Y-axis: S/I; Z-axis: M/L). A dashed line indicates no significant trend. An upward (downward) arrow indicates a linearly increasing (decreasing) trend. An upward (downward) curved arrow indicates a quadratically increasing (decreasing) trend.

With a rotational perturbation of 6 degrees in both directions about the Z-axis (i.e., specimen rotation in the sagittal plane), the most sensitive parameter again was MLATa, overestimating the standard values by 145.3% for positive rotational perturbations and by 87.0% for negative rotational perturbations (Table 4). The second most sensitive parameter was MDA, underestimating the standard values by about 56.3% for positive rotational perturbations, but overestimating by about 19.3% for negative rotational perturbations. Another parameter was MDV, showing a sensitivity of about 20% with different error values in response to positive and negative rotational perturbations. None of the parameters were sensitive to rotational perturbations about the Y-axis, i.e., in the transverse plane, where all sensitivities were less than 10% (Table 4). Linear or quadratic trends of changes in most parameters in response to rotational perturbations about the X- and Y-axes were detected, but only a small number of parameters showed significant trends when subject to perturbations about the Z-axis (Table 4).

Most of the 14 parameters analyzed showed very good reliability with ICC values ranging from 0.81 to 1.0: TiAL (0.99), TiSR (0.95), APG (0.97), APA (0.97), MTiTh (0.95), TaAL (0.92), TaR (0.95), TiW (0.97), MalW (0.91) and MLATa (0.89). Parameters with good reliability were MLATi (0.78), MDA (0.79) and MDV (0.65). The only parameter with a moderate reliability was TaW (ICC = 0.55).

The linear regression equations derived from data in the standard pose performed quite well for poses of the specimen models that were perturbed within 3 degrees in the three individual rotational components. After correction with the linear regression equations (Table 3), the 2D measurements errors of all the parameters were greatly reduced with residual errors less than 10% of the 3D measurements, except for MLATa (14.76%) (Table 5). Pose errors up to 6 degrees in the three individual rotational components were also reduced in most of the parameters, except for MDA (35.96%), MDV (12.09%), TaW (-14.07%) and MLATa (29.11%) (Table 5).

**Table 5:** Correction of 2D measurements using linear regression equations

		**Perturbed pose (3 Deg)**		**Perturbed pose (6 Deg)**	
		**Error (%)**	**Corrected error (%)**	**Error (%)**	**Corrected error (%)**
**Sagittal Plane**	*Tibia Part*				
	TiAL	2.17	-1.02	4.41	1.12
	TiSR	3.17	-0.05	4.87	1.59
	APG	3.51	-0.14	13.34	9.42
	APA	2.69	2.69	8.40	8.40
	MTiTh	2.91	-1.66	5.56	0.25
	MDA	31.36	8.42	65.25	35.96
	MDV	25.43	8.63	33.46	12.09
	*Talus Part*				
	TaAL	-8.57	1.95	-5.92	0.58
	TaR	7.08	1.10	8.35	1.84
**Frontal Plane**	*Tibia part*				
	TiW	4.97	-1.85	6.87	-0.23
	MalW	2.37	0.54	3.54	1.66
	MLATi	2.21	2.07	2.74	2.60
	*Talus part*				
	TaW	-10.61	-8.78	-16.36	-14.07
	MLATa	29.80	14.76	50.54	29.11

*Error = 100% × (2D - 3D)/3D.

Mean residual errors of 2D measurements of the morphological parameters made on two sets of DRR images produced from 3D poses with random errors in the three individual rotational components for up to 3 degrees and 6 degrees, respectively, before and after corrections using the linear regression equations derived from data in the standard pose.

## Discussion

The current study aimed to compare the measurements of ankle morphology in the standard pose using 3D CT images with those using 2D images, and to determine the relationships between 2D and 3D measurements using linear regression analysis; to determine the sensitivity of the 2D measurements to mal-positioning of the ankle during imaging; to quantify the repeatability of the 2D measurements under simulated positioning conditions involving random errors; and to evaluate the performance of the linear regression equations in predicting 3D measurements from 2D measurements. In the standard pose, six out of fourteen 2D morphological parameters were not significantly different from the 3D gold standard values, and most of the parameters were highly correlated between 2D and 3D measurements. Most 2D measurements were not sensitive to rotational perturbations, except for TaW, MLATi and MLATa to perturbations about the X-axis, and MDA, MDV and MLATa to perturbations about the Z-axis. Most of the 2D measurements were repeatable when subjected to randomized perturbations about each anatomical axis, except for TaW that had only moderate repeatability. The linear regression equations between the 2D and 3D measurements were shown to be effective in correcting the errors in the 2D morphometric measurements, which will be helpful for improved estimation of the morphometric parameters for clinical purposes.

In the standard pose for imaging, most morphometric parameters measured using 2D images were highly correlated to the gold standard values defined by the 3D CT data. This was expected because in the standard imaging pose the morphometric parameters measured from the 2D images were related to the 3D data via the principle of perspective projection with only little variation among subjects. Nonetheless, significant differences between 2D and 3D measurements did exist in several morphometric parameters. On the other hand, for those few morphometric parameters, namely MTiTh, MDV and TaR, that had poor to moderate correlations with the 3D gold standard, no significant differences were found between their 2D and 3D measurements. This indicates that if the imaging is done in the standard pose, the 2D measurements can be regarded as good representations of the parameters as those measured using 3D CT data. However, in a clinical setting it may be unfeasible to position the ankle reliably in the standard pose because patients have different profiles and sizes of the thigh, shank and the calcaneus that may affect the pose of the foot/ankle complex.

For measurements made on the M/L radiographs (i.e., in the sagittal plane), most of the parameters were found not to be affected by rotational errors about the three axes. The only sensitive parameters were MDA and MDV, which have important implications for TAA because these parameters are related to determining the optimal cutting level of the bone and the relevant instrumentation in ankle arthroplasty in order to preserve the healthy bone of the tibia and fibula as much as possible [4]. For measurements made on the A/P radiographs (i.e., in the frontal plane), most of the parameters were found not to be affected by rotational errors about the three axes. The only sensitive parameters were MLATi, MLATa and TaW. The first two parameters were small in value. Therefore, any small changes due to small rotational errors in the frontal and/or sagittal planes would be a large proportion of the original value. In contrast, for the same positive positioning error, TaW considerably underestimated the standard values by up to 31.2%. This underestimation was equivalent to an error of 8 mm in Chinese adults [20], which would be a major problem for sizing the total ankle prosthesis, the maximal range of which is only about 10 mm. This was further worsened by its reduced reliability compared to most of the other parameters. These results suggest that pre-surgical evaluations based on TaW should be made with caution so as to avoid inappropriate selection of the sizes of the TAA prosthesis because the value of the trochlea tali width (TaW) relates directly to the size of the talar component [27].

Since it is unrealistic to expect the ankle to be positioned in the standard pose reliably in a clinical setting, one possibility is to try to reduce the 2D measurement errors given that 2D and 3D measurements of most of the morphological parameters were found to be correlated linearly. The linear regression equations obtained in the current study were shown to be effective in correcting the errors in the 2D morphometric measurements, especially for perturbation less than 3 degrees about the three individual axes. However, when the positioning errors became too large, such as up to 6 degrees, parameters sensitive to the axial perturbations, namely MDA, MDV, TaW and MLATa, were difficult to correct. This is mainly because the linear regression equations derived from the data in the standard pose were less effective in describing the 2D and 3D relationship accurately. Therefore, care should be exercised in positioning the ankle in order to minimize the positioning errors in clinical practice. With physically minimized positioning errors, the current linear regression equations would be helpful for improved estimation of the morphometric parameters for clinical purposes.

In the current study, CT-based computer simulation with DRRs was preferred over the direct use of simple X-ray radiographs because the latter would need more manual involvement, increasing uncertainties and errors in the measurements during imaging. For example, an accurate positioning device that allows the angular perturbations about the three axes of the coordinate system for each specimen with different sizes and morphology would be needed. Manual identification of the bony landmarks separately on the specimen and on the X-ray image would also be required. Nonetheless, since measurements based on planar radiographs are still widely used for the diagnosis of diseases and pre-surgical evaluations of the ankle joint in orthopedic practices, it is essential to establish some knowledge of the errors or uncertainties in the radiograph-based measurements. The current results identified the parameters whose 2D measurements were sensitive to rotational pose errors during imaging. Linear regression equations were also obtained for correcting 2D measurements for clinical use. Further studies are needed to quantify the sensitivity, reliability and correction equations for the parameters when subject to errors in the ankle joint positions (e.g., plantar- and dorsiflexion), as well as translational pose errors such as when the source of the X-ray is not focused on the reference points used for positioning during imaging. Further studies may also include reliability analysis under real life conditions to account for intra- and inter-observer variability that have been suggested to be the main source of artifacts in clinical practice [28].

## Conclusions

In the standard pose for imaging, most ankle morphometric parameters measured using 2D images were highly correlated to the gold standard values defined by the 3D CT data, with some morphological parameters not significantly different from the gold standard values. For measurements made on the M/L images, the only parameters sensitive to rotational pose errors were MDA and MDV, which had important implications for determining the optimal cutting level of the bone during TAA surgery. For measurements made on the A/P images, TaW underestimated the standard values by up to 31.2% with only a moderate reliability, suggesting that pre-surgical evaluations based on TaW should be made with caution so as to avoid inappropriate selection of the sizes of the TAA prosthesis. The linear regression equations of the relationship between 2D and 3D measurements will be helpful for correcting the errors in 2D morphometric measurements for clinical applications.

## Competing interests

The authors declare that they have no competing interests.

## Authors’ contributions

CCK prepared the specimens, participated in the CT data collection and analysis, and drafted the manuscript. HLL developed the software programs, performed data analysis and helped draft the manuscript. TWL participated in the study design, methodological development, interpretation of the results and drafting the manuscript. CCL helped develop the software programs and interpret the results. AL participated in data interpretation and the revision of the manuscript. MYK participated in the study design and CT data collection. HCH participated in the study design and revision of the manuscript. All authors read and approved the final manuscript.

## References

[B1] Henricson A Skoog A Carlsson Å The Swedish Ankle Arthroplasty Register: an analysis of 531 arthroplasties between 1993 and 2005 Acta Orthop 2007 12 5 569 574 10.1080/17453670710014248 17966014

[B2] Yalamanchili P Neufeld S Lin S Total ankle arthroplasty: a modern perspective Curr Pract Orthop Surg 2009 12 2 106 110 10.1097/BCO.0b013e31819b02d2

[B3] Leardini A Moschella D Dynamic simulation of the natural and replaced human ankle joint Med Biol Eng Comput 2002 12 2 193 199 10.1007/BF02348124 12043800

[B4] Stagni R Leardini A Ensini A Cappello A Ankle morphometry evaluated using a new semi-automated technique based on X-ray pictures Clin Biomech 2005 12 3 307 311 10.1016/j.clinbiomech.2004.11.009 15698704

[B5] Pyevich MT Saltzman CL Callaghan JJ Alvine FG Total ankle arthroplasty: A unique design: Two to twelve-year follow-up J Bone Joint Surg-Am Vol 1998 12 10 1410 1420 9801209

[B6] Conti SF Wong YS Complications of total ankle replacement Clin Orthop 2001 12 105 114 11603658 10.1097/00003086-200110000-00011

[B7] Myerson MS Mroczek K Perioperative complications of total ankle arthroplasty Foot Ankle Int 2003 12 1 17 21 12540076 10.1177/107110070302400102

[B8] Kofoed H Sørensen TS Ankle arthroplasty for rheumatoid arthritis and osteoarthritis J Bone Joint Surg-Br Vol 1998 12 2 328 332 10.1302/0301-620X.80B2.8243 9546471

[B9] Leardini A Catani F Giannini S O'Connor JJ Computer-assisted design of the sagittal shapes of a ligament-compatible total ankle replacement Med Biol Eng Comput 2001 12 2 168 175 10.1007/BF02344799 11361242

[B10] Leardini A O'Connor JJ Catani F Giannini S Mobility of the human ankle and the design of total ankle replacement Clin Orthop 2004 12 7 39 46 15241142 10.1097/01.blo.0000132246.26172.b7

[B11] Buechel FF Pappas MJ The New Jersey low-contact-stress knee replacement system: Biomechanical rationale and review of the first 123 cemented cases Arch Orthop Trauma Surg 1986 12 3 197 204 3753173 10.1007/BF00435480

[B12] Anderson T Montgomery F Carlsson Å Uncemented STAR total ankle prostheses: Three to eight-year follow-up of fifty-one consecutive ankles J Bone Joint Surg-Am Vol 2003 12 7 1321 1329 12851358

[B13] Shimmin AJ Walter WL Esposito C The influence of the size of the component on the outcome of resurfacing arthroplasty of the hip: A review of the literature J Bone Joint Surg-Br Vol 2010 12 4 469 476 10.1302/0301-620X.92B4.22967 20357319

[B14] Bolland BJRF Culliford DJ Langton DJ Millington JPS Arden NK Latham JM High failure rates with a large-diameter hybrid metal-on-metal total hip replacement: Clinical, radiological and retrieval analysis J Bone Joint Surg-Br Vol 2011 12 5 608 615 10.1302/0301-620X.93B5.26309 21511925

[B15] Kempson GE Freeman MAR Tuke MA Engineering considerations in the design of an ankle joint Biomed Eng 1975 12 5 166 171 1125359

[B16] Calderale PM Garro A Barbiero R Biomechanical design of the total ankle prosthesis Eng Med 1983 12 2 69 80 10.1243/EMED_JOUR_1983_012_020_02 6683677

[B17] Conn KS Clarke MT Hallett JP A simple guide to determine the magnification of radiographs and to improve the accuracy of preoperative templating J Bone Joint Surg-Br Vol 2002 12 2 269 272 10.1302/0301-620X.84B2.12599 11922371

[B18] Gourineni PVRKV Knuth AE Nuber GF Radiographic evaluation of the position of implants in the medial malleolus in relation to the ankle joint space: Anteroposterior compared with mortise radiographs J Bone Joint Surg-Am Vol 1999 12 3 364 369 10.2106/00004623-199903000-00008 10199274

[B19] Tochigi Y Suh JS Amendola A Pedersen DR Saltzman CL Ankle alignment on lateral radiographs. Part 1: Sensitivity of measures to perturbations of ankle positioning Foot Ankle Int 2006 12 2 82 87 16487458 10.1177/107110070602700202 PMC2274959

[B20] Kuo C-C Lu H-L Leardini A Lu T-W Kuo M-Y Hsu H-C Three-dimensional computer graphics-based ankle morphometry with computerized tomography for total ankle replacement design and positioning Clin Anat 2013 in press doi:10.1002/ca.22296 10.1002/ca.22296 23960000

[B21] Fessy MH Carret JP Béjui J Morphometry of the talocrural joint Surg Radiol Anat 1997 12 5 299 302 10.1007/BF01637597 9413076

[B22] Lin CC Lu TW Shih TF Tsai TY Wang TM Hsu SJ Intervertebral anticollision constraints improve out-of-plane translation accuracy of a single-plane fluoroscopy-to-CT registration method for measuring spinal motion Med Phys 2013 12 3 031912 10.1118/1.4792309 23464327

[B23] Hadwiger M Kniss JM Rezk-salama C Weiskopf D Engel K Real-Time Volume Graphics 2006 London, UK: A. K. Peters

[B24] Chen C-C Lin C-C Chen Y-J Lu T-W Huang C-Y Accuracy assessment of a marker-cluster registration method for measuring temporomandibular kinematics using cone-beam computerized tomography with fluoroscopy J Med Biol Eng 2013 12 443 448 10.5405/jmbe.1155

[B25] Shrout PE Fleiss JL Intraclass correlations: Uses in assessing rater reliability Psychol Bull 1979 12 2 420 428 18839484 10.1037//0033-2909.86.2.420

[B26] Altman DG Practical Statistics for Medical Research 1991 London: Chapman & Hall/CRC

[B27] Giannini S Leardini A O'Connor JJ Total ankle replacement: Review of the designs and of the current status Foot Ankle Surg 2000 12 2 77 88 10.1046/j.1460-9584.2000.00202.x

[B28] Saltzman CL Brandser EA Berbaum KS DeGnore L Holmes JR Katcherian DA Teasdall RD Alexander IJ Reliability of standard foot radiographic measurements Foot Ankle Int 1994 12 12 661 665 10.1177/107110079401501206 7894638

